# EARLY-LIFE EXPOSURE TO THE CHINESE FAMINE OF 1959–1961 AND LATER-LIFE HEALTH: EARLY LIFE AS A CRITICAL PERIOD

**DOI:** 10.1093/geroni/igac059.1930

**Published:** 2022-12-20

**Authors:** Mengling Cheng, Nicolas Sommet, Daniela Jopp, Dario Spini

**Affiliations:** University of Lausanne, Lausanne, Vaud, Switzerland; University of Lausanne, Lausanne, Vaud, Switzerland; University of Lausanne, Lausanne, Vaud, Switzerland; University of Lausanne, Lausanne, Vaud, Switzerland

## Abstract

Barker’s fetal origins hypothesis and the critical period theory suggest that early life events have long-term health effects. However, evidence of the famine exposure in early life and its effects on health in later life is scarce and inconsistent. To explore the effects of early-life exposure to the Chinese famine of 1959-1961 on later-life multimorbidity, we performed Poisson growth curve models using CHARLS Life History 2014 and CHARLS 2011-2018 (42,775 observations from 12,060 respondents). Our analyses revealed two findings. First, there was an overall detrimental effect of the early-life famine exposure on multimorbidity, although there was no effect of severity of famine exposure. Second, there was no overall interaction between famine exposure and life stages, although a more parsimonious model suggested that the detrimental effect of famine exposure was more pronounced in earlier life stages than in later life stages. Findings suggest that early life is a critical period in the life course and provides developmental origins of health and disease in later life.

